# Supplementary material for: Structure of puromycin-sensitive aminopeptidase and polyglutamine binding
Source: PLoS One. 2023 Jul 13;18(7):e0287086. doi: 10.1371/journal.pone.0287086 (PMC10343166; doi:10.1371/journal.pone.0287086)
Supplement: S1 Table — (DOCX) [file pone.0287086.s001.docx]

**Table S1: Data for graphs in Fig. 10**

| Data for Fig. 1A, Ala-βNA series at three dyn A(1-17) concentrations | | | | |
| --- | --- | --- | --- | --- |
| 1/[Ala-βNA] (1/μM) | 1/v (1/nmol/min/ug) | | | |
|  | 0.0 μM  dyn A(1-17) | 0.75 μM  dyn A(1-17) | 1.0 μM  dyn A(1-17) |  |
|  |  |  |  |  |
| 0.01 | 0.107 | 0.144 | 0.244 |  |
| 0.02 | 0.126 | 0.244 | 0.348 |  |
| 0.03 | 0.150 | 0.348 | 0.495 |  |
| 0.04 | 0.166 | 0.466 | 0.647 |  |
| 0.05 | 0.199 | 0.520 | 0.773 |  |
| 0.06 | 0.223 | 0.621 | 0.960 |  |
| 0.08 | 0.271 | 0.755 |  |  |
|  |  |  |  |  |
| Data for Fig. 1B, Ala-βNA series at four polyQ peptide concentrations | | | | |
| 1/[Ala-βNA] (1/μM) | 1/v (1/nmol/min/ug) | | | |
|  | 0.0 μM polyQ | 1.0 μM polyQ | 2.6 μM polyQ | 5.2 μM polyQ |
| 0.01 | 0.114 | 0.129 | 0.156 | 0.170 |
| 0.02 | 0.126 | 0.165 | 0.197 | 0.240 |
| 0.03 | 0.137 | 0.199 | 0.254 | 0.337 |
| 0.04 | 0.149 | 0.242 | 0.283 | 0.368 |
| 0.05 | 0.167 | 0.271 | 0.330 | 0.434 |
| 0.06 | 0.180 | 0.288 | 0.391 | 0.495 |
| 0.08 | 0.213 | 0.320 | 0.466 |  |
|  |  |  |  |  |
| Data for Fig. 1C, dyn A(1-17) inhibition of PSA^WT^ | | | | |
| [dyn A(1-17)] (μM) | 1/v (1/nmol/min/ug) | | | |
| 0.0 | 0.216 | 0.217 | 0.219 |  |
| 0.2 | 0.258 | 0.261 | 0.250 |  |
| 0.4 | 0.296 | 0.310 | 0.297 |  |
| 0.6 | 0.343 | 0.364 | 0.345 |  |
| 0.8 | 0.417 | 0.397 | 0.405 |  |
| 1.0 | 0.461 | 0.502 | 0.497 |  |
| 1.2 | 0.574 | 0.517 | 0.508 |  |
|  |  |  |  |  |
| Data for Fig. 1D, dyn A(1-17) inhibition of PSA^F433A^ | | | | |
| [dyn A(1-17)] (μM) | 1/v (1/nmol/min/ug) | | | |
| 0.0 | 12.094 | 11.530 | 11.031 |  |
| 1.0 | 15.072 | 14.917 | 14.691 |  |
| 2.0 | 17.807 | 18.247 | 17.001 |  |
| 3.0 | 20.352 | 20.438 | 20.745 |  |
| 4.0 | 23.540 | 22.647 | 22.933 |  |
| 5.0 | 26.765 | 25.631 | 25.100 |  |
|  |  |  |  |  |
| Data for Fig. 1E, polyQ inhibition of PSA^WT^ | | | | |
| [polyQ] (μM) | 1/v (1/nmol/min/ug) | | | |
| 0.0 | 0.166 | 0.169 | 0.167 |  |
| 0.7 | 0.204 | 0.196 | 0.206 |  |
| 1.3 | 0.242 | 0.245 | 0.251 |  |
| 2.0 | 0.269 | 0.303 | 0.307 |  |
| 2.6 | 0.334 | 0.345 | 0.356 |  |
| 3.3 | 0.383 | 0.394 | 0.435 |  |
|  |  |  |  |  |
| Data for Fig. 1F, polyQ inhibition of PSA^F433A^ | | | | |
| [polyQ] (μM) | 1/v (1/nmol/min/ug) | | | |
| 0.0 | 12.667 | 13.681 | 12.903 |  |
| 6.6 | 18.994 | 20.089 | 20.799 |  |
| 13.2 | 34.831 | 35.892 | 36.130 |  |
| 26.3 | 62.515 | 62.605 | 62.247 |  |
| 39.5 | 83.956 | 93.908 | 81.445 |  |
| 52.6 | 90.401 | 103.746 | 92.121 |  |
